# Chronic Health Crises and Emergency Medicine in War-torn Yemen, Exacerbated by the COVID-19 Pandemic

**DOI:** 10.5811/westjem.2021.10.51926

**Published:** 2022-02-28

**Authors:** Mohammed Alsabri, Luai M. Alsakkaf, Ayman Alhadheri, Jennifer Cole, Frederick M. Burkle

**Affiliations:** * Brookdale University Hospital and Medical Center, Department of Pediatrics, Brooklyn, New York, USA; † Al Thawra Modern General Hospital, Department of Emergency Medicine, Sana’a City, Yemen; ‡ McLaren Oakland/Michigan State University, Department of Emergency Medicine, Pontiac, Michigan, USA; § Royal Holloway University of London, Department of Health Studies, Egham Hill, Egham, Surrey TW20 0EX, United Kingdom; ¶ Harvard University & T.H. Chan School of Public Health, Harvard Humanitarian Initiative, Cambridge, Massachusetts, USA

## Abstract

**Introduction:**

Much of Yemen’s infrastructure and healthcare system has been destroyed by the ongoing civil war that began in late 2014. This has created a dire situation that has led to food insecurity, water shortages, uncontrolled outbreaks of infectious disease and further failings within the healthcare system. This has greatly impacted the practice of emergency medicine (EM), and is now compounded by the coronavirus disease 2019 (COVID-19) global pandemic.

**Methods:**

We conducted a systematic review of the current state of emergency and disaster medicine in Yemen, followed by unstructured qualitative interviews with EM workers, performed by either direct discussion or via phone calls, to capture their lived experience, observations on and perceptions of the challenges facing EM in Yemen. We summarize and present our findings in this paper.

**Results:**

Emergency medical services (EMS) in Yemen are severely depleted. Across the country as a whole, there are only 10 healthcare workers for every 10,000 people – less than half of the WHO benchmark for basic health coverage – and only five physicians, less than one third the world average; 18% of the country’s 333 districts have no qualified physicians at all. Ambulances and basic medical equipment are in short supply. As a result of the ongoing war, only 50% of the 5056 pre-war hospitals and health facilities are functional. In June 2020, Yemen recorded a 27% mortality rate of Yemenis who were confirmed to have COVID-19, more than five times the global average and among the highest in the world at that time.

**Conclusion:**

In recent years, serious efforts to develop an advanced EM presence in Yemen and cultivate improvements in EMS have been stymied or have failed outright due to the ongoing challenges. Yemen’s chronically under-resourced healthcare sector is ill-equipped to deal with the additional strain of COVID-19.

## INTRODUCTION

Of Asia’s 48 countries, Yemen is the 12^th^ largest in terms of total area (527,970 square kilometers) and the 20^th^ in terms of population (30 million).^1^ Over the past three decades, many religious, geographical, historical, and economic obstacles have caused harsh divisions in the country, resulting in a civil war that started in 2014 and continues today. This conflict has led to an unprecedented humanitarian crisis, including extensive violations of humanitarian law and the Geneva Convention by combatants from all factions.^2^ Attacks and airstrikes that began in March 2015 have included strikes on hospitals and medical facilities – an egregious violation of international, humanitarian, and human rights laws.^3^ This includes, according to conservative documentation by the World Health Organization (WHO), at least 142 attacks on medical facilities in Yemen between 2015– 2019.^4^

The conflict in Yemen has so far claimed the lives of 233,000 people. according to the United Nations Office for the Coordination of Humanitarian Affairs, 5 As a result of the ongoing war, only 50% of the 5056 pre-war hospitals and health facilities are functional.^6^ The conditions of even those hospitals are nowhere near full potential: they are in dire need of essential equipment and proper funding.^6^ The ongoing blockade by land, air, and sea has limited the extent of secure access to international aid, proper supplies, and humanitarian support, creating the world’s most extreme humanitarian crisis.^3^ The many years of prolonged instability has led to the proliferation of armed militias and militant groups which, despite several attempts at a ceasefire, have continued and escalated their aggression. Most disheartening is that the situation is not in the hands of Yemenis themselves but is perpetrated by both international and local powers. The continued instability caused by the pandemic enables terrorist groups to continue their operations,^7^ threatens financial collapse,^8^ and causes the absence of government protections.^9^

Even before the coronavirus 2019 (COVID-19) pandemic reached Yemen, widespread repercussions of the ongoing war had proved devastating: Yemen is one of the poorest countries not only in the Middle East but in the entire world.^10^ The population has been left to fend for itself as Yemenis face severe hunger, outbreaks of infectious diseases, unattended acute and chronic diseases, and lack of basic healthcare infrastructure. Healthcare workers lack equipment and supplies.^6^ Most recently, the COVID-19 pandemic has added to this already devastating humanitarian crisis. We report here on the practice of emergency medicine (EM) in Yemen, which is operating in a chronic crisis mode, exacerbating and increasing the unprecedented new challenges brought about by the COVID-19 pandemic. Where not explicitly cited to another source, observations in this report come from the first-hand experience of the authors.

## METHODS & DISCUSSION

In this report we review the status of EM in Yemen and describe the challenges faced by its practitioners. The information was gathered through systematic reviews of PubMed, Ovid, and governmental websites using the terms “emergency medicine.” “disaster medicine,” “emergency medical services,” “challenges,” “COVID-19,” and “Yemen.” Furthermore, we conducted multiple, unstructured interviews with EM workers, performed by either direct discussion or via phone calls, to capture their lived experience and their observations on and perceptions of the challenges facing EM in Yemen. We summarize and present our findings in the text and tables below. The participants were general practitioners (GP) and specialists who were identified by convenience sampling from different hospitals in Yemeni cities, and who have more than five years’ experience working in EM. One author is a departmental vice chair, co-founder and first president of the Yemeni Association of Emergency Medicine and Disasters and has extensive knowledge of EM in the historic capital city, Sana’a. Two of the authors are emergency physicians who are involved in the development of EM residency training programs in Yemen. Their experiences provide unique insight into the challenges faced by a health system under crisis.

**Population Health Research Capsule**

What do we already know about this issue?

*Yemen has been severely impacted by civil war and now COVID-19. This has impacted its emergency medical services and left many hospitals under-resourced.*

What was the research question?

*What is the state of emergency medical care in Yemen including prehospital care and emergency ambulance services?*

What was the major finding of the study?

*Yemen’s under-resourced healthcare sector is ill-equipped to deal with the additional strain of COVID-19.*

How does this improve population health?

*By highlighting the lack of resources and paucity of training opportunities, we illustrate the need for the international healthcare community to support their Yemeni colleagues.*

### Challenges for Emergency Medicine

One of the key challenges facing Yemen is an acute shortage of healthcare workers. For every 10,000 people, there are only 10 healthcare workers in Yemen, which is less than half of the WHO benchmark for basic health coverage,^11^ and only five physicians, less than one third the world average, compared with 26 per 10,000 in Saudi Arabia and 20 in Oman, Yemen’s neighbouring countries.^12^ Practicing as an emergency physician in Yemen is uniquely difficult, profoundly impacting one’s overall perspective as a practicing physician. A shortage of supplies presents insurmountable challenges to conditions that would be treatable under normal conditions, such as otitis media,^13^ headache,^14^ and nonspecific chest pain.^15^ Such experiences have forced the specialty to reconsider and reshape how EM is practiced in Yemen. This has resulted in a process based on rapid decision-making in sub-optimal conditions across several domains, for example, managing challenging arrangements for transportation: trauma victims and acutely ill patients are often transported to hospital by bystanders who use their own, non-medical, vehicles.^8^

### Current Emergency Medical Services and Paramedic Status

Yemen lacks any formal prehospital emergency medical services (EMS).^8^ Ambulances available from the Ministry of Health, and those in public and private sector health facilities, are used solely for the sake of inter-hospital transportation, with little coordination. Medical helicopters are only available for military services and some oil production facilities. Even if a medical center does have an ambulance, that does not mean the vehicle is always available or can provide emergency care. Ambulance vehicles usually lack proper equipment, with the exception of some ambulances donated by non-governmental organizations (NGO)^16^ and a few owned by the Ministry of Health. Transport-to-hospital policies are either not in place or not easily activated due to the lack of financial support. When mass casualty incidents occur in Yemen – a common occurrence due to the consequence of the ongoing conflict – victims are often transported to hospital in private cars by people present at the scene.^8^

Emergency call numbers often fail during medical emergencies. Even if calls are connected, requests for an ambulance are almost always rejected and the caller is advised to bring the patient in by any other available means. This situation has resulted from the lack of applicable policies and an overarching sense of insecurity prevalent among healthcare workers: ambulance staff fear direct assaults on the ambulance and on themselves personally,^8^ as ambulances have been the target of airstrikes during the ongoing war. The Yemen Red Crescent Society has 60 ambulance vehicles; however, they are used only for inter-hospital or inter-healthcare facility transport, reporting directly from their headquarters in Sana’a, the historic, former capital of Yemen. The exact scale of the problem is difficult to quantify due to the challenges of carrying out research in Yemen: while frontline medical staff highlight a shortage of ambulances and vehicles it has not been possible to determine how many are in the country even after contacting key persons in the Yemeni healthcare authorities.^16^

In the city of Aden, the current capital, there are no dedicated ambulances for COVID-19 patients, although transport did improve when Médecins Sans Frontières (MSF) (Doctors Without Borders) took charge of the al-Amal facility’s management following an escalation of the number of cases of COVID-19 in the governorate in May 2020.^17^ Similar desperate situations have been witnessed across Yemen.

The country also lacks qualified paramedics. Nineteen paramedics graduated in 2014 from the Higher Institute of Health Sciences in Sana’a, with a one-year diploma in emergency and ambulance services; however, no graduates or formally trained paramedics have been certified since the beginning of the war, nor is there a formal training pathway approved or available for paramedics.^18^ Thus, when compared to standard levels of care in other countries, Yemen’s EMS services might be considered functionally non-existent, particularly when compared with those in neighboring Saudi Arabia, which has more than 20 training centers across the country, a four-year training curriculum based on the North American model, and two-year EM fellowships.^17^,^18^

### Status of Training of Emergency Clinicians

Overall, emergency departments (ED) in Yemen primarily depend on general practitioners (GP) as the main emergency care clinicians, irrespective of their experience. There are more GPs available, and they are more affordable than trained EM specialists, as their annual income in some regions starts as low as $800 United States dollars (USD). Recently, there have been attempts in larger hospitals to employ more EM specialists. This has been augmented further by the Ministry of Public Health and Population (MOPHP) directive in 2020 that mandated hospitals hire EM specialists; in reality, however, these ambitions have been far from possible to realize considering the small number of available specialists in the country. It is, however, a step in the right direction toward increasing the value of EM and developing more EDs across the country.

In practice, GPs face even more obstacles than emergency physicians. Hospitals without emergency physicians lack the holistic approach in their care for patients who present to the ED. While the physicians have considerable experience in treating such patients, neither they nor their facilities are well-suited to providing emergency care. This impacts the care of patients as well as the experience of the GPs. At the patient level, for example, a trauma patient who comes in with multiple acute and complex issues might not be managed holistically and systematically, as there would more likely be a focus on a single injury such as traumatic brain injury or major limb fracture. Such a situation may delay other critical diagnoses for the same patient. General practitioners may struggle to manage their patients, to prepare their departments for receiving such patients, and assess patient flow.

It is common to see improperly planned and poorly supplied EDs: the GPs working in the EDs as well as EM specialists face ongoing challenges. Only one EM training center in Yemen, Al-thawra Modern General Hospital (TMGH) in Sana’a, is recognized by the Yemeni Board for Medical Specialisation and the Arab Board of Health Specializations (ArBHS). It is a Level I trauma center and a tertiary referral hospital with advanced healthcare services. This designation means that it has the capability to comprehensively treat all types of injury and serves as a regional resource for patients of all ages. Its facilities include a kidney transplant center, dialysis center, stroke center, cardiac center with catheterization laboratory, and an advanced cardiac surgery center. The ED normally sees around 320,000 unique visits annually. It is the busiest hospital in Yemen and is considered the most prestigious, serving over 30 million people: the entire population of the country.

Al-thawra Modern General Hospital is ranked highly as an ArBHS-approved training center for multiple medical specialties including EM, regionally as well as locally. While EM trainees from countries such as Iraq, Syria, and Somalia choose to train at the facility, few of them choose to follow a career in Yemen. Graduates of the four-year EM medical training center – the only one in Yemen – who successfully pass their board examination receive the Arab Board of Emergency Medicine certification and are licensed by the Yemeni MOPHP and the Yemeni Physicians Syndicate. These graduates can work independently as consultants or attending physicians. As of May 2021, an estimated 220 physicians have finished their EM residency programme in Yemen. However, very few of these graduates still work in Yemen: most have moved to work throughout the world, mainly in other regions of the Arab Gulf.^8^

Board-certified emergency physicians tend to leave the country due to the war, the humanitarian crisis, and overall worsening poverty; they migrate to nearby countries in search of higher wages and an overall sense of greater respect for the specialty and security for themselves and their families. This pattern is not uncommon; such an exodus of qualified staff is common in conflict-affected regions and is recorded in many other countries, including Iraq^20^ and Syria^21^ to name but two. The EM specialists who have remained in Yemen continue to endure mediocre facilities, poor administration, and lack of proper compensation. They are usually overworked.^18^,^22^ In Yemen, EM specialists and consultants annually earn between $6,000–15,000 USD with an annual average of $10,000 USD. For junior emergency physicians, the annual income starts around $4400 USD. In comparison, in the Kingdom of Saudi Arabia the annual income is $70,000–165,000 USD.^18^ Nurses are similarly underpaid. This leads to a heavy dependence on newly graduated GPs: they are less of a financial burden and are more willing to work for lower wages but are not usually required to have any specialized training or to have attended any specific EM courses. Because of the marked shortage and urgent need for board-certified emergency physicians, most EDs and trauma centers are led by GPs.

### Working in an Overwhelmed and Limited Environment

Yemen’s poor economic state and the ongoing civil war has put a toll on the availability of critically important medical supplies. Those that are available are often poorly maintained, hindered by lack of finances. Respondents to our survey reported seeing broken radiograph or computed tomography machines, some of which have been inoperable since before the start of the civil war. Emergency physicians reported working without critical supplies and instruments and having to ask the relatives of patients suffering from cardiac arrest to buy epinephrine and endotracheal tubes to ensure their treatment. Benzodiazepines, vasopressors, and intravenous (IV) lines are not always available in EDs. Personal protective equipment (PPE) was scarce even before the COVID-19 pandemic. Previously, physicians faced the 2009 H1N1 outbreak with only basic surgical masks, and many reported having to buy their own PPE including N95 masks.^23^ Some physicians have intubated diphtheria patients with only basic surgical masks to protect them.

Antivenoms, antidotes for common toxicities such as digoxin and opioids, and other basic medicines such as activated charcoal are rarely available. Physicians reported having to sometimes call small strikes in the ED to force the hospital to provide the bare minimum of life-saving drugs and supplies, including epinephrine and IV cannulas, as supplies are not readily available. In other instances, physicians reported demanding that pharmaceutical companies provide such supplies, using their own contacts at the local level. Respondents reported wealthier physicians bringing with them as many critical medications as they can personally obtain; they would often ask a patient’s relatives to try to locate substitutes from outside of the hospital, in order to provide prompt interventions and prevent further delays to essential treatment. The damaged infrastructure in Yemen adds to the challenges. Emergency physicians report having to intubate with defective laryngoscopes and without any light at all. They resort to using flashlights from mobile phones or small lighters to provide illumination while intubating patients on the floor. Monitors and direct current (DC) shock devices in the cardiopulmonary (CPR)/resuscitation rooms are poorly or under-maintained: one respondent reported using a DC shock device as a cardiac monitor in the CPR/resuscitation room due to crowding and defective monitors.

Many Yemenis have adopted carrying weapons as part of their regional dress code. Hospital security has historically mandated gun-free zones; however, with the widespread absence of hospital security, relatives of patients enter the CPR/resuscitation room with machine guns while their relatives are resuscitated, to protect themselves from attacks on hospitals, which unfortunately is a reality in Yemen. Many physicians in EDs report instances (infrequent but often enough to impact their experience) where they have been threatened by armed relatives while trying to treat their patients. In the vast majority of triage rooms across the country, no formal system or protocol is used to process patients apart from shifting shocked patients through to the red zone.

### Challenges of Infectious Disease Management During the COVID-19 Pandemic

The sparsity of medical professionals is evident across Yemen; around 18% of the country’s 333 districts do not have any physicians.^24^ The country’s instability and deaths among the medical community have resulted in an increasing exodus of medical personnel, and the ongoing instability has also led to disruptions in higher education, resulting in a decline in skilled medical professionals as few new ones come through the system.^5^ The COVID-19 pandemic has only exacerbated this already existing problem. At the beginning of the COVID-19 outbreak, the complete lack of PPE and safety measures put ED staff and community health workers at great risk.^25^ Healthcare workers also face increasing threats and attacks from family members of COVID-19 patients.^26^ The human toll of one physician’s death extends to the entire community, which will be left without access to the healthcare he or she would have provided. Due to the shortage of physicians to consult and lack of easy access to physicians, some COVID-19 patients initially misdiagnosed their symptoms and sought treatment with medications they could buy over the counter, putting pharmacists and pharmacy staff at a particularly high risk of infection from COVID-19 at a time when communities increasingly depend on those pharmacists and pharmacy staff due to the lack of emergency physicians and functioning hospitals, creating a vicious circle.^26^

Yemenis lack sufficient access to clean water and sanitation.^27^ The ongoing war, the displacement of millions of people, and seasonal flooding create the ideal conditions for the spread of infectious and communicable diseases. In hospitals, infection control measures are often impossible to implement: many hospitals completely or partially lack sinks and soap.^26^ There is no national workforce strategy to employ more epidemiologists, and there is a shortage of technical staff required for evidence-based field investigation or active surveillance.^28^ In 2019 alone, there were more than 760,000 suspected cases of cholera, 25,000 cases of dengue, 1600 cases of diphtheria, and nearly 10,000 suspected cases of measles.^26^ The country is still suffering a seemingly never-ending cholera epidemic that has led to over two million cases and over 3000 deaths,^26^ the worst documented epidemic in the history of the disease.^29^,^30^ Widespread dengue fever and chikungunya infections have made the diagnosis of COVID-19 even more difficult in areas without proper or adequate testing. Patients with such diseases often present with very similar symptoms to those of COVID-19, but they require different treatments.^26^ With the lack of a functioning government, points of entry into the country, including the airports in Aden and Seiyun, as well as the ports of Alwadia’ah, Sharowrah, and Algaithah, do not have the necessary isolation stations for suspected or confirmed COVID-19 cases. There is little opportunity to enact or uphold the standards set by the International Health Regulations.

### Challenges to the Patient-Clinician Relationship

According to the UN, in 2004 the overall literacy rate for the Yemeni population 15 and older was 54.1%.^31^,^32^ While this number has been slowly improving, other aspects of education, such as health literacy, are of great concern as it exacerbates delays in seeking care, leads to poorer health status, and compounds lack of knowledge about medical conditions, particularly when healthcare professionals are hard to access. Physicians often face judgment by patients’ relatives in the case of a complication or a death, and reports of assaults on physicians or other healthcare workers across the country are not uncommon. The COVID-19 pandemic has brought about an escalation of these extremes from the relatives of suspected COVID-19 patients. In April 2020, a physician in Aden was threatened at gunpoint when he could not admit a patient to his overwhelmed facility due to lack of equipment, medical staff, and available beds.^33^ A combination of decades of conflict, illiteracy, and the ready availability of weapons to civilians is a toxic mix in which medical professionals struggle to practice.

### Worsening Economic Crisis

The situation is not helped by the economic decline Yemen has been facing for many years. Fuel exports are drying up and the Yemeni rial (YR) is rapidly depreciating against foreign currencies. The COVID-19 crisis has led to a further drop in the demand for and prices of Yemen’s fuel exports, leading to even greater depreciation of the rial. For example, in 2014, $1 USD equated to 214 YR. In 2021, $1 USD equated to 910 YR in Aden and 600 YR in Sana’a. The economic crisis is worsening even as other nations have made major cuts in funding for humanitarian assistance as they struggle to support their own economies during the pandemic. Inside Yemen, this is complicating the COVID-19 response: a severe fuel shortage in the northern governorates hinders the transportation of medical supplies and the powering of generators.

In addition to facing the heightened risk of contracting COVID-19 while treating patients in hospitals, many medical staff have not been paid a salary in up to two years.^26^ For the last several years, the WHO had been paying incentives to thousands of medical staff throughout Yemen as a short-term measure.^26^,^34^,^35^ However, as of mid-April 2020, the same week that the COVID-19 crisis started in Yemen, the WHO cut incentive payments for 10,000 healthcare workers in the country. Both the danger of COVID-19, coupled with the cuts to salaries and the removal of incentive payments, has led to a wave of resignations. Lise Grande, the UN humanitarian coordinator in Yemen, states that it is “a funding crisis of gargantuan proportions.”^36^

### Gaps in Disaster Preparedness

The 2011 Yemeni Revolution served as an opportune time to study the preparedness of the country in the face of disasters. A comparative study was conducted between 2011–2013^18^ using the WHO checklist evaluation, but the study found that no significant progress had been made by Sana’a city hospitals concerning hospital disaster preparedness during that time. The checklist included nine key components: command and control; communication; safety and security; triage; surge capacity; continuity of essential services; human resources; logistics and supply management; and post-disaster recovery.^37^ The study was conducted in 11 hospitals in the capital city of Sana’a, indicating that these hospitals have remained poorly equipped to meet the needs of the patient population during times of disasters. Ongoing disasters have since rendered the system progressively weaker: they have cost the country dearly, shifting what little budget there is toward relief, instead of preparedness and development.

One may legitimately question how a country can prepare for disaster if it is constantly in a state of disaster? In the time of siege and war, airstrikes have only made matters worse. Housing has suffered greatly from direct airstrikes. The catastrophic impact of war has led to further cuts in MOPHP budgets. Local media footage shows the impacts of these budget cuts as victims of such incidents are evacuated using non-ambulance vehicles, which emphasize the lack of out-of-hospital disaster preparedness. According to the UN, the lack of fuel has also contributed to the absence of ambulances.^38^

In 2018 health professionals in Yemen were surveyed to determine their knowledge of and attitude toward disaster preparedness.^18^ This included 531 healthcare professional responses. While the concept of disaster preparedness was not unheard of among participants, only a third had a sound understanding of the management of disasters. Among all the different staff members, nursing staff were the least knowledgeable, even when compared to health administrators. Of the study respondents, 41% stated they had never received instruction on disaster preparedness at any time during their academic studies or professional careers.^18^

Unfortunately, disaster preparedness in Yemen has not received the necessary attention from the authorities or the international community and will only be recognized when proper planning, funding, and budgeting exists. Disaster preparedness must be incorporated into informal training programs for all healthcare professionals including emergency medical technicians and other hospital staff. The development of EMS itself will aid in the development of disaster preparedness. The military, the Ministry of Interior, the MOPHP, firefighters, and others must all be involved in such planning and training, as the issue is multi-sectoral and cannot be the sole responsibility of the MOPHP.

### COVID-19 Management Prognosis

Epidemiologists have estimated that without drastic action, COVID-19 will spread “faster, more widely and with deadlier consequences” in Yemen than in other countries. Jens Laerke, spokesman for the UN Office for the Coordination of Humanitarian Affairs stated that the COVID-19 pandemic is severely exacerbating the humanitarian crisis in the country, which was already the world’s most severe.^39^ Acute challenges include “[u]nsafe shelters, persistent migration and displacement, lack of essential medicines, inadequate food and insufficient access to safe drinking water, suppressed immunity among the malnourished population” and that many areas of the country lack the lower limit of hygiene standards. These are optimal conditions for the rampant spread of infectious diseases,^9^ making preventing and combating infectious diseases more challenging in Yemen than elsewhere.

After reporting its first case of COVID-19 on the 10th April 2020, by June Yemen had a 27% mortality rate of Yemenis who were confirmed to have COVID-19 – more than five times the global average and among the highest in the world at that time, as physicians were forced to practice with the minimal levels of safety.^26^ “We are re-using personal protective equipment because we don’t have enough,” said Dr. Khairil Musa, a MSF ICU specialist in the city of Aden in January 2021.^40^ As a result of the ongoing situation, some hospitals closed because they were worried about contamination, or because of lack of essential supplies needed to protect the health of workers.^41^ Médecins Sans Frontières, among other medical relief organizations working in Yemen, are desperately calling for more PPE to be imported to the country. Without corrective actions in place, Yemen’s people and healthcare systems may completely crumble.

## DISCUSSION

In this special report we bring together first-hand accounts that are often complex and difficult to collect. It is based on these internal insights that the complexity of the matters at hand can begin to be understood or at the very least appreciated. This report offers some of the first documented lived experiences of Yemeni physicians operating on the frontline of the COVID-19 pandemic. Previous studies have not gone into such depth; this report paves the way for even more first-hand accounts to be documented. Potential future research can begin addressing the individual ailments of the collective disease. The EM community is consistently at the forefront of a plethora of societal and humanitarian adversities: what troubles a society and people is usually evident first and foremost in the EDs to which they present. One of our goals in writing this overview was to provide insight to the global EM community into the extent and severity of the challenges, epidemics, conflicts, and disasters that affect the EM world.

Corrective actions to address these challenges include both short-term reactive measures as well as longer term preventative measures. Some Yemeni physicians and highly educated officials who have studied and worked outside of Yemen, have a robust knowledge base and a solid understanding of the disaster management and preparedness protocols used worldwide. Having this knowledge, however, is not enough. Resources are required for tangible change. The solutions to Yemen’s current situation must target the challenges from multiple angles. Solutions must come from education and training programs dedicated to filling in the gaps throughout the short- and long-term interventions. Solutions might include fostering community and neighborhood involvement and invoking assistance from volunteers and activists of all ages to pilot smaller improvement projects. Lastly, a systems approach to emergency and disaster management is needed.

Lessons can also be identified from disaster relief programs in other conflict-affected regions. Literature from the field of health systems research in fragile and conflict affected states, from countries including Syria,^21^,^42^ Iraq,^43^ Sri Lanka,^44^ Afghanistan^45^ and Sierra Leone,^46^,^47^ points to many successful approaches to facilitating long-term health system recovery. In Syria, for example, an extremely effective relief solution has been the initiation and implementation of locally led projects supported by expatriate philanthropic and charitable funding. This has ensured that needs can be swiftly identified and prioritized on the ground in a rapidly changing context.^42^,^48^ Professional associations link in-country medics with an expatriate diaspora that can provide training, help to establish new facilities through donations of finances, equipment and expertise, including facilitating e-health and telemedicine consultations,^48^ and thus help to identify local gaps by facilitating remote links with overseas researchers.^49^ Weak local research capacity creates challenges with attracting external funding and collaborators, particularly long term, while rapidly changing conditions mean that what research is conducted is soon outdated,^45^ requiring innovative and flexible solutions^46^ driven from the ground up: remote connection provides one such solution.

Secondly, systems need to be supported for systematic and robust data collection and surveillance,^45^ including for breaches of human rights and humanitarian law as well as epidemiology, as the former can be used to improve advocacy and to keep both policymakers who are subject to rapid turnover and NGO workers up to date with situations on the ground.^49^ Provision for surveillance systems can often take second place to provision for services,^45^ but their breakdown can have dire consequences for early identification of disease outbreaks, particularly when coupled with concurrent breakdown of vaccination programs.^47^ A third area of action should foster support for, or implementation of, programs for psychological support and resilience, particularly for those understandably traumatized by their experiences^50^ and for children.^42^

Thirdly, facilities in conflict zones face challenges with water and electricity supply, and medical equipment supplies and personnel. Lack of water and electricity are symptomatic of wider challenges with maintaining infrastructure^44^,^51^ and harder to solve unilaterally, but the availability of medical equipment and supplies can be improved by stockpiling better reserves in safe zones inside the country, to facilitate supply chain resilience. Such an approach has proved highly successful in Iraq^43^ and can help to ease challenges such as needing to transfer patients from one facility to another due to shortage of materials rather than lack of ability to perform the necessary procedures.^44^

Fourthly, the issue of qualified medical staff leaving conflict zones for safer jobs elsewhere is not unique to Yemen: it has been well documented in Iraq,^20^ Syria,^21^ and Nigeria^50^ among many other countries. One solution is for the government, local authorities, and humanitarian aid to provide better security to healthcare facilities and workers, as lack of such security presents challenges not only to retaining local staff but also to attracting NGO and humanitarian support, and to enabling such people to move around the country freely once they are in country.^45^

Finally, part of the challenge is to ensure that whatever measures and solutions are implemented, they are planned with the long-term perspective kept firmly in mind, so that any short-term humanitarian relief can transition smoothly to in-country sustainability and from there to strengthening the healthcare sytem, which will have lasting impact.^52^

A takeaway from this report is the need for standardized assessments of Yemen’s current situation. Change can only occur when the challenges confronted are truly understood and evaluated. Moving forward, effort should be allocated to using two different, but equally effective, systemic resources. The first is the WHO toolkit for assessing healthcare-system capacity for crisis management.^53^ This evaluation toolkit provides a standardized hospital emergency response checklist. The second resource is a popular model and organizational tool implemented in the EM world: the US Federal Emergency Management Agency’s “Four Phases of Emergency Management” consists of mitigation, preparedness, response, and recovery (Figure). This tool is holistic and can continuously be referenced. It can be used before and after events, as well as in anticipation of future events and challenges.

## CONCLUSION

As emergencies and disasters continue to occur, so does the sense of urgency and emphasis on improving reactive and future proactive measures. The Yemeni people have never been immune from suffering disasters caused by a wide spectrum of societal ills as well as the ravages of nature. The state of the nation, including ongoing conflicts and a poor healthcare system superimposed on the current COVID-19 global pandemic, has exacerbated an already crippled emergency medicine infrastructure. David Beasley, executive director of the UN Security Council, states that this is the UN agency’s biggest emergency and has made an appeal for the surrounding Gulf states to financially help save the lives of Yemenis. In this global public health emergency, only immediate attention from the international community will salvage the current health crisis.

## Figures and Tables

**Figure:**
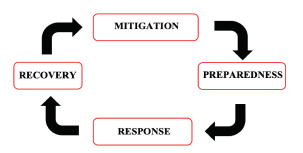
Four phases of emergency management per the US Federal Emergency Management Agency.
